# Effect of Transportation on Cultured Limbal Epithelial Sheets for Worldwide Treatment of Limbal Stem Cell Deficiency

**DOI:** 10.1038/s41598-018-28553-0

**Published:** 2018-07-12

**Authors:** O. A. Utheim, T. Lyberg, J. R. Eidet, S. Raeder, A. Sehic, B. Roald, E. Messelt, M. F. de la Paz, D. A. Dartt, T. P. Utheim

**Affiliations:** 1 Department of Medical Biochemistry, Oslo University Hospital, Oslo, Norway; 2 Department of Ophthalmology, Oslo University Hospital, Oslo, Norway; 3 Norwegian Dry Eye Clinic, Oslo, Norway; 4 Department of Oral Biology, Faculty of Dentistry, University of Oslo, Oslo, Norway; 5 Department of Pathology, Oslo University Hospital, Oslo, Norway; 6 Institut Universitari Barraquer, Universitat Autonoma de Barcelona, Barcelona, Spain; 7 Schepens Eye Research Institute/Massachusetts Eye and Ear Infirmary, Department of Ophthalmology, Harvard Medical School, Boston, MA USA

## Abstract

Limbal stem cell deficiency can be treated with transplantation of cultured human limbal epithelial cells (LEC). It can be advantageous to produce LEC in centralized labs and thereafter ship them to eye clinics. The present study used transport simulations of LEC to determine if vigorous shaking during transport altered the viability, morphology and phenotype during a 4 day-long storage of LEC with a previously described serum-free storage method. Inserts with LEC cultured on amniotic membranes were sutured to caps inside air-tight containers with generous amounts of 4-(2-hydroxyethyl)-1-piperazineethanesulfonic acid (HEPES)-buffered minimal essential medium (MEM). The containers were distributed among the following testing conditions: 6 hours with full containers, 36 hours with full containers, 36 hours with container three quarters full of medium, and 36 hours with container full of medium containing a shear-protecting agent (Pluronic-F68). Compared to stored, but non-transported controls, no statistically significant changes in viability and immunohistochemical staining were observed. The epithelial sheets remained intact. However, an air-liquid interface in the containers reduced the number of desmosomes and hemi-desmosomes compared to the controls. In conclusion, cultured LEC sheets appear to endure vigorous shaking for at least 36 hours if the container is full.

## Introduction

The surface of the cornea contains tissue-specific stem cells that maintain homeostasis and regeneration of the corneal surface. Most literature supports the concept that these stem cells are located circumferentially in the periphery of the cornea, the limbal region^1,2^. A variety of diseases (e.g. Stevens Johnsons syndrome, aniridia), injuries (e.g. chemical or thermal burns) and external factors (e.g. infections, including trachoma) may damage the limbal stem cells, resulting in either partial or total (360°) limbal stem cell deficiency (LSCD). In 1997, LSCD was for the first time treated by transplantation of *ex vivo* cultured limbal stem cells^3^. Since then more than 1000 transplantations have been performed to treat LSCD^4^. Nevertheless, the treatment remains limited to a few centres of expertise^5^. Ever stricter regulations for cell therapy promote centralization of culture units^6^, which call for reliable and practical transportation strategies^7^.

Storage of cultured LEC in a sealed container for some days, increases flexibility for the surgeon in the planning of operations, and enables quality testing and transportation of the LECs prior to surgery. The importance of establishing good methods for storage and transportation has been highlighted following the recent European Medicine Agency’s (EMA) recommendation of approving LEC therapy in Europe^8^. This approval is a major step for regenerative medicine in Europe and limbal regenerative therapy in particular as it represents the first recommendation by EMA for any stem cell therapy in Europe. The approval also reflects that corneal regenerative medicine is in the forefront of regenerative medicine.

Several reports have been published on the various aspects of storage of cultured LEC^5,9–15^, while transportation of epithelial sheets for ocular surface reconstruction has been studied to a limited extent. In 2014, Vasania *et al*. tested an in-house designed transportation container for cultured conjunctival epithelial cell sheets on human amniotic membrane (HAM), with viable, intact epithelial sheets upon arrival and good post-operative outcome for pterygium surgery^16^. Oie *et al*. created a sterile, temperature-stable container for culture dishes that kept air pressure at atmospheric levels^17^. Rabbit LEC and cultured human oral mucosa were successfully transported in the container for 5 hours in an airplane. However, weaker expression of zonula occludens -1 (ZO-1) was observed after the transport, suggesting that the transport may cause a reduction in intercellular adherence and barrier function.

Transport is different from storage in the sense that the tissue is exposed to movement, that unlike other environmental factors, cannot be eliminated by a sealed transport container. Our research group recently developed a serum- and xenobiotic-free storage method of 4–7 days for human limbal epithelial cells (HLEC) cultured on HAM^5^ that could serve as the basis for transporting cultured tissue. As rigorous shaking may occur during transport both on the road and in the air, we used the previously described storage method^5^ and simulated extreme transport conditions followed by a storage period. Duration of the transport simulation, the presence or absence of an air-liquid interface inside the storage bottles and the addition of a shear force protecting agent to the medium were tested using HLEC sheets that were stored, but not transported as the control. We found that transport simulations of up to 36 hours appeared not to be critical to the viability, ultrastructure and phenotype of HLECs with a completely filled container.

## Results

### Donor Characteristics of Cultured Cells

Limbal rings of three male donors, aged 71, 80 and 82 years, were harvested at Barraquer Ophthalmology Centre in Spain 12–18 hours post mortem, and shipped to Oslo on day 3, 4 and 6 post mortem. Time from harvest to culture was 10 to 11 days.

### Effect of Transportation on Viability of LEC Sheets

**Figure 1:**
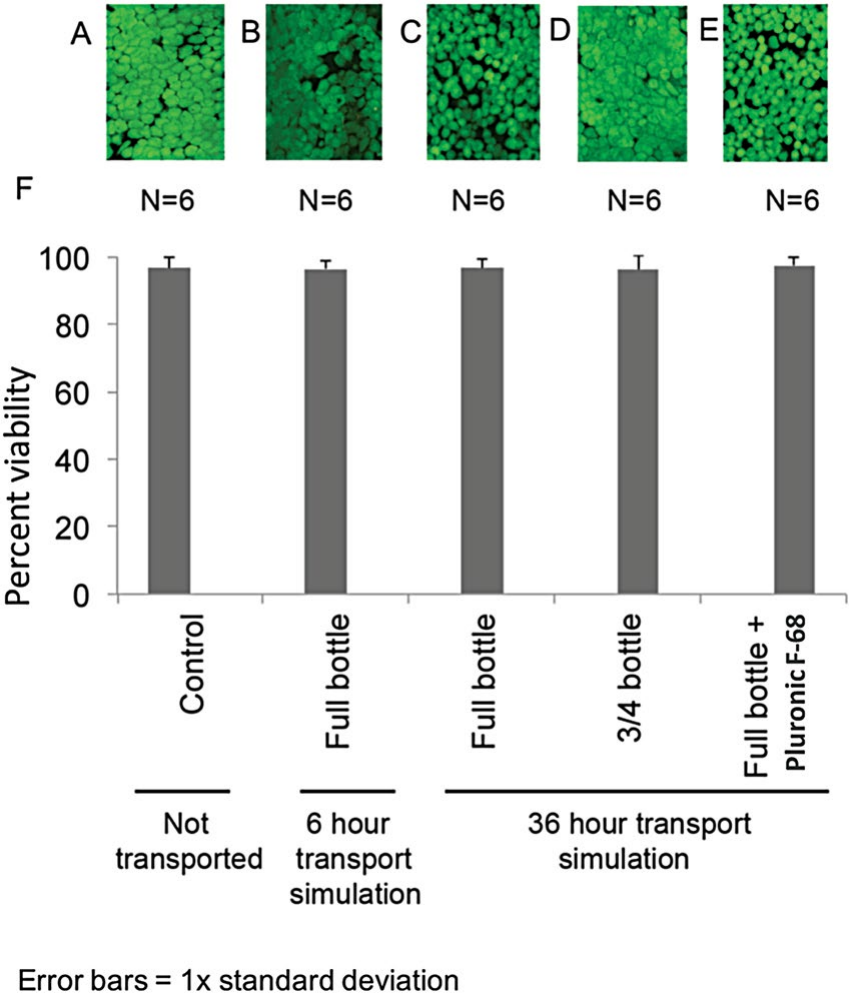
Viability of LECs after Transport Simulation and/or Storage. (**A**–**E**) Laser confocal micrographs of the basal layer of cultured human limbal epithelial cell (LEC) sheets after viability staining. The photos represent LECs after (**A**) storage, but no transport simulation (control), (**B**) transport simulation in bottles full of medium followed by storage (**C**) 36-hour transport simulation in full bottles + storage (**D**) 36-hour transport simulation in ¾ filled bottles + storage (**E**) 36-hour transport simulation in bottles full of medium added the surfactant Pluronic F-68. Original magnification x250. (**F**) Bar chart illustrating the percentage of viable cells in LEC sheets for the respective groups. N = number of cultures. Error bars = 1 standard deviation.

Cellular viability was calculated from fluorescent images of LEC basal layers after treatment with a live/dead staining kit as shown in Fig. 1A–E. Mean amounts of viable cells as a percentage of the total number of cells are presented in Fig. 1F, based on viability data as presented in Supplementary Data S1. Viability for the stored, but not transported, control group was 97 with standard deviation (SD) 3.1%. After transport simulation and storage, the viability of the LEC sheets remained high (between 89% and 100%) for all LEC sheets. There were no statistically significant changes compared to the non-transported control, regardless of transport time (6 versus 36 hours), amount of container filling (three quarter versus completely filled containers) and composition (with or without Pluronic F-68) of HEPES-MEM based medium (Fig. 1). Hence, the viability of the LEC sheets remained high for all transport simulations.

### Effect of Transportation on Morphology of Cells in LEC Sheets

**Figure 2:**
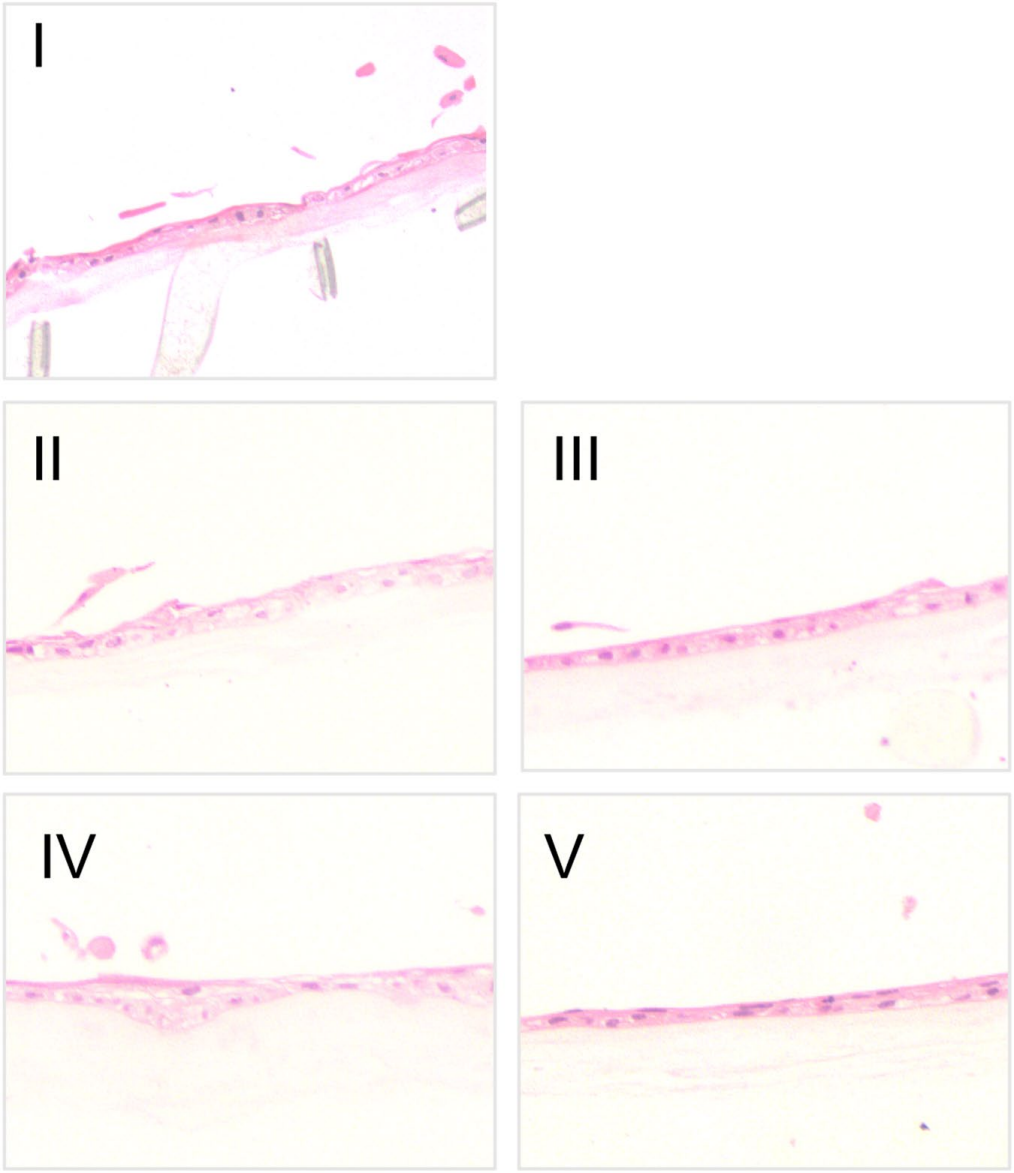
H&E-sections of LECs after Transport Simulation and/or Storage. Hematoxylin & Eosine (H&E)-stained sections of stratified, non-keratinized limbal epithelial cell (LEC) sheets cultured on amniotic membranes representative for each experimental group in the study. Some detached cells are seen above the epithelial surface of both non-transported and transported sheets. (**I**) Represents stored, but not-transported cultures, (**II)** transport simulation for 6 hours in full bottle, (**III**) transport simulation for 36 hours in full bottle, (**IV**) transportation simulation for 36 hours in bottle three quarters filled with medium, (**V**) transportation simulation for 36 hours in full bottle with medium added the surfactant Pluronic-F68. Original magnification x10.

LEC sheet morphology was evaluated based on light microscopy Hematoxylin & Eosin (H&E) sections and transmission electron microscopy (TEM) micrographs from samples of every stored and/or transported LEC sheet. Representative examples of H&E sections are presented in Fig. 2. Mean central thickness of LEC sheets per experimental group measured on H&E sections and mean number of cell layers calculated from TEM micrographs are presented in Supplementary Fig. S1. All H&E and TEM thickness data are available in Supplementary Data S2.

The LEC sheets demonstrated epithelia well attached to the HAM. Some detached cells were observed above the epithelial surface of both non-transported and transported sheets. No differences in shedding of cells were observed between the non-transported control group and the four different transport conditions (Fig. 2).

Mean central thickness per sheet measured from H&E section images was 15.6 SD 6.0 *μ*m for the non-transported control (n = 6), 13.5 SD 5.0 *μ*m for the 6-hour transport group with full container (n = 5), 20.7 SD 9.8 *μ*m for the 36-hour transport group with full container (n = 6), 9.7 SD 2.0 *μ*m for the 36-hour transport group with three quarters full container (n = 5), and 20.5 SD 9.3 *μ*m for the 36-hour transport group with full container added Pluronic F-68 (n = 6). There were no statistically significant differences between the groups (Supplementary Fig. S1).

The number of cell layers based on TEM micrographs varied between 2 and 6 with a mean of 3.23 SD 0.89 (n = 5) for the stored, but non-transported control group. There were no significant differences between the non-transported control and transported groups (Supplementary Fig. S1).

In summary, the transport simulations did not cause any reduction of cell layers and LEC thickness compared to a stored, but non-transported control.

### Effect of Transportation on Inter- and Intracellular Adherence of Cells

**Figure 3:**
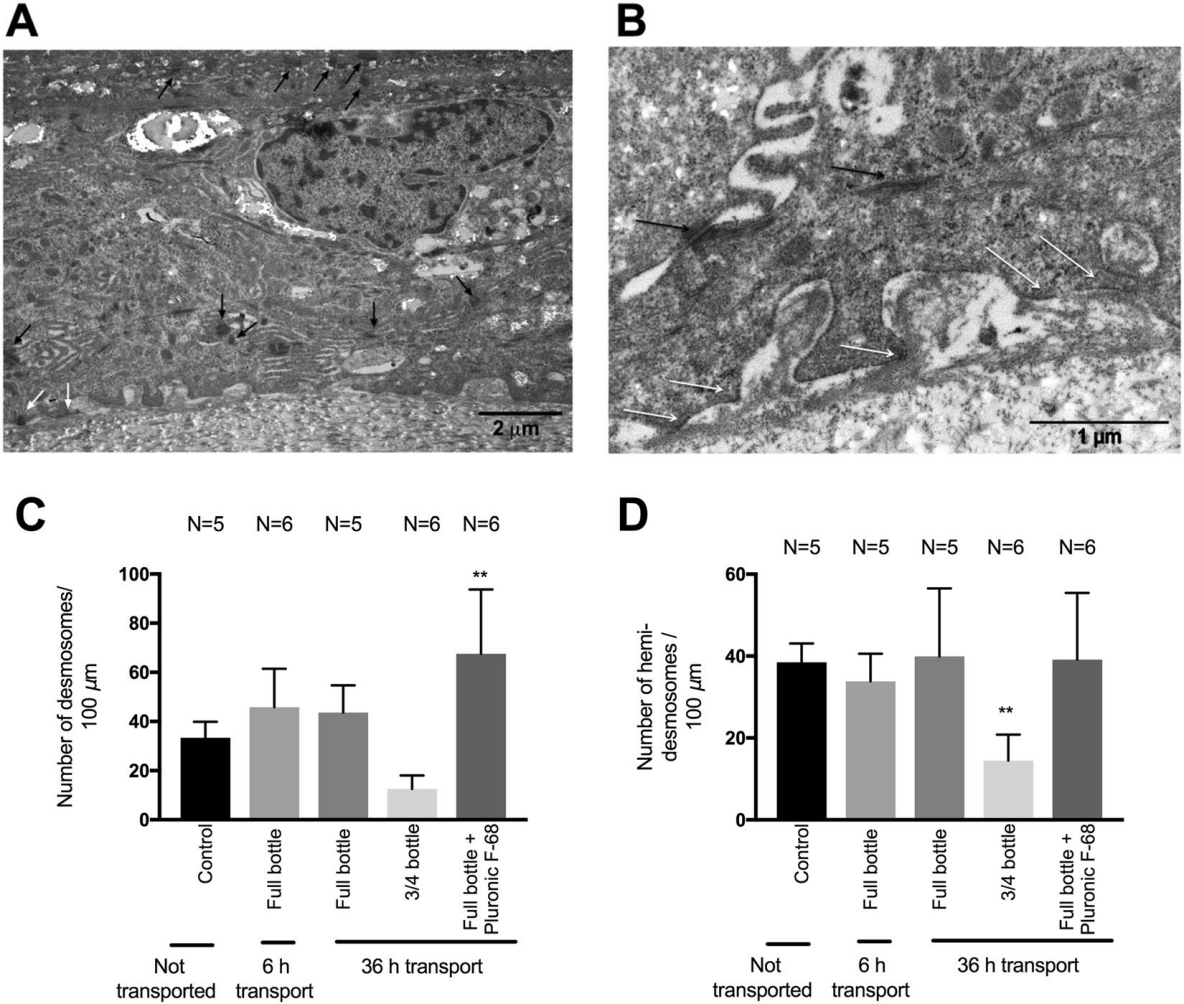
Desmosome and hemidesmosome imaging and analysis. Transmission electron microscopy (TEM) micrographs at 9700× magnification (**A**) and 33 000× magnification (**B**) of cultured human limbal epithelial cells (LECs) that underwent storage, but not transport simulation (control). The cells are tightly connected to the amniotic membrane with hemi-desmosomes (white arrow head) and to neighboring cells with desmosomes (dark arrow head). Bar charts illustrating the mean numbers of desmosomes (**C**) and hemidesmosomes (**D**) per 100 *μ*m of cultured human limbal epithelium not subjected to transport simulation (control) versus transport simulation under various conditions (6 hours versus 36 hours, HEPES-MEM with or without the surfactant Pluronic F-68, and bottle filled to the cap with medium versus ¾ full bottle). Error bars = 1 standard deviation. **p < 0.01 compared to the control. N = Number of cultures.

Changes in inter- and intracellular adherence after transport simulation were assessed by the numbers of desmosomes and hemi-desmosomes in TEM micrographs. TEM images of LEC sheets demonstrating desmosomes and hemi-desmosomes are presented in Fig. 3A and B. The underlying data for the analyses of desmosomes and hemi-desmosomes are presented as Supplementary Data S3.

Mean numbers of desmosomes for every group are presented in Fig. 3C. For stored, but not transported cultured LEC sheets (n = 5), mean number of desmosomes was 33 SD 6.5 per 100 *μ*m of epithelium (Fig. 3C). After transport simulation, the lowest mean number of desmosomes was 13 SD 5.5 per 100 *μ*m of epithelium for 36 hours in the three quarters full container (p = 0.11). The highest mean number of desmosomes was 68 SD 26 per 100 *μ*m of epithelium after transport simulation for 36 hours in full container with medium added the surfactant Pluronic-F68, and significantly higher than the non-transported control (p < 0.01).

Figure 3D demonstrates the mean number of hemi-desmosomes per group. For stored, but not transported cultured LEC sheets (n = 5), mean number of hemi-desmosomes was 38 SD 4.6 per 100 *μ*m of epithelium (Fig. 3D). After transport simulation, the mean number of hemi-desmosomes after 36 hour-transport in three quarters full container was significantly reduced to 14 SD 6.4 per 100 *μ*m of epithelium (p < 0.01). For all other transport simulation groups, mean numbers of hemi-desmosomes were unchanged compared to the non-transported control group, with p-values of more than 0.99.

In summary, a reduction of cell junctions was observed in the transport simulation group with an air-liquid interface in the containers (significant for hemi-desmosomes, approaching, but not reaching significance for desmosomes). For containers completely filled with medium, the cell junctions were unaffected of transport time (6 versus 36 hours). The additive Pluronic F-68 gave a significantly increased amount of desmosomes, but not hemi-desmosomes, compared to the non-transported control.

### Effect of Transportation on Phenotype, Proliferation and Apoptosis of LEC Cell Sheets based on Immunohistochemical Analyses

**Figure 4:**
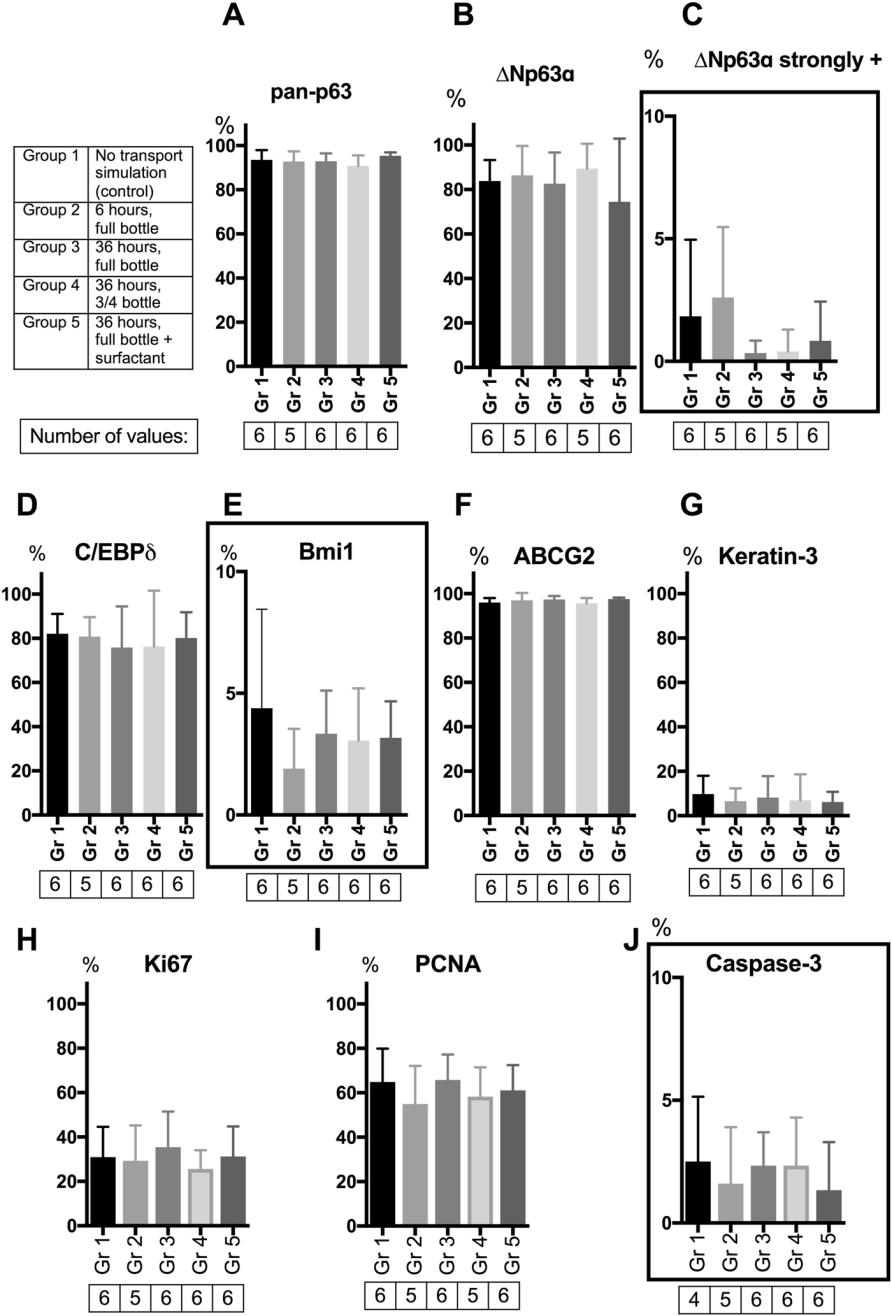
Expression of Immunohistochemical Markers for LECs after Transport Simulation and/or Storage. Bar charts demonstrating mean positivity of immunohistochemical markers for human limbal epithelium not subjected to transport simulation (control) versus transport simulation under various conditions (6 hours versus 36 hours, HEPES-MEM with or without the surfactant Pluronic F-68, and bottle filled to the cap with medium versus ¾ full bottle). The markers are (**A**) pan-p63, (**B**) ∆Np63α, (**C**) ∆Np63α strongly positive, (**D**) C/EBP∂, (**E**) Bmi-1, (**F**) ABCG2, (**G**) Keratin 3, (**H**) Ki67, (**I**) PCNA, and (J) Caspase-3. Error bars = 1 standard deviation. N = Number of cultures. Framed bar charts have y-axis scale 0–10 % while the non-framed bar charts have y-axis scale 0–100 %.

Variations in immunohistochemical expression between the experimental groups are displayed as bar charts in Fig. 4. Examples of the immunohistochemical sections are presented in Supplementary Fig. S2 with one representative micrograph per experimental group for each of the immunohistochemical markers. Variations in marker expression between basal and supra-basal layers are presented in detail in Supplementary Table S1, and the complete immunohistochemical data are available in Supplementary Data S4. In the following paragraphs the results are presented briefly.

The staining pattern of the transcription factor p63 (pan-p63), a marker for immature cells^18,19^, was nuclear (Supplementary Fig. S2), extensive throughout both basal and supra-basal layers (Supplementary Table S1), and with a mean expression of 94 SD 4% of cells for the non-transported control group (Fig. 4A). There were no statistically significant differences in expression of pan-p63 for any of the transport simulation groups compared to the control (Fig. 4A).

The ∆Nα isotype of p63 is a marker of holoclone-forming keratinocytes and early transit amplifying cells^18,19^. The ∆Np63α-staining was granular, and persistent it nuclei and cell membranes in basal and supra-basal cells of the epithelium (Supplementary Table S1, Supplementary Fig. S2). Mean ∆Np63α expression was 84 SD 9% for the control group (Fig. 4B). There were no statistically significant differences in expression of ∆Np63α for any of the transport simulation groups compared to the control (Fig. 4B).

Occasionally, some of the ∆Np63α-positive cells stood out with densely stained cell nuclei (for example in Supplementary Fig. S2B IV) as opposed to the more scattered, granular staining throughout the LEC sheets (for example in Supplementary Fig. S2B I, II, III and V). The densely stained nuclei were considered strongly positive to ∆Np63α, and counted and analysed separately. Mean of strongly positive ∆Np63α was 2 SD 3% for the non-transported control group (Fig. 4C). There were no statistically significant differences in strong expression of ∆Np63α for any of the transport simulation groups compared to the control (Fig. 4C).

The staining pattern of the holoclone-associated, putative stem cell marker CCAAT/enhancer binding protein delta (C/EBP∂)^18^ was nuclear and extensive (Supplementary Fig. S2) throughout both basal and supra-basal layers (Supplementary Table S1). The mean expression for the control was 82 SD 9% of the cells (Fig. 4D). There were no statistically significant differences in expression of C/EBP∂- for any of the transport simulation groups compared to the control (Fig. 4D).

The Polycomb complex protein Bmi1 (Bmi1) is previously shown to mark mitotically quiescent, putative limbal stem cells^18^. Very few nuclei were positive for Bmi1 in the present study (Supplementary Fig. S2). Occasionally, there were some Bmi1-staining in cell membranes and basally towards the amniotic membrane (Supplementary Fig. S2). Only cells with nuclear staining were counted as positive for Bmi1. The staining occurred mostly, but not exclusively, in the basal layers of the LEC sheets (Supplementary Table S1). The mean expression for the control (stored, but non-transported LEC sheets) was 4 SD 4% of the cells (Fig. 4E). There were no statistically significant differences in expression of Bmi1- for any of the transport simulation groups compared to the control (Fig. 4E).

The membrane transporter protein ATP-binding cassette sub-family G member 2 **(**ABCG2) is a marker of immature cells^20^. The staining for ABCG2 was extensive throughout both nuclear and cell membranes (Supplementary Fig. S2) in both basal and supra-basal layers (Supplementary Table S1). Mean expression for the control (stored, but non-transported LEC sheets) was 96 SD 2% of the cells (Fig. 4F). There were no statistically significant differences in expression of ABCG2 for any of the transport simulation groups compared to the control (Fig. 4F).

Keratin 3 (K3) is a marker of terminally differentiated corneal epithelium^2^. K3 was expressed in cell membranes in supra-basal layers in a patchy manner in both non-transported and transported groups (Supplementary Fig. S2, Supplementary Table S1). The majority of LEC sheets had a K3-expression of 5% or less of the cells, and the maximum expression of K3 detected per LEC sheet was 30% of the cells. Mean K3-expression for non-transported LEC sheets was 10 SD 8% of the cells (Fig. 4G). There were no statistically significant differences in expression of K3 for any of the transport simulation groups compared to the control (Fig. 4G).

The staining of the proliferation marker Ki67^21^ was equally distributed in the cell nuclei (Supplementary Fig. S2) in both basal and suprabasal layers (Supplementary Table S1). Mean Ki67-expression for non-transported LEC sheets was 31 SD 14% of the cells (Fig. 4H). There were no statistically significant differences in expression of Ki67 for any of the transport simulation groups compared to the control (Fig. 4H).

Staining for proliferating cell nuclear antigen (PCNA)^22,23^ was also evenly distributed in cell nuclei (Supplementary Fig. S2) in both basal and suprabasal layers (Supplementary Table S1). Mean positivity of PCNA was 65 SD 15% for the control group (Fig. 4I). There were no statistically significant differences in expression of PCNA for any of the transport simulation groups compared to the control (Fig. 4I).

Staining for Caspase 3; a DNA cleavage enzyme expressed in apoptotic cells^24^, is demonstrated in LEC cells in a previous study of the group^5^. In the present study, the Caspase 3-staining was mostly negative for all groups (Supplementary Fig. S2, Supplementary Table S1). Mean positivity of Caspase 3 was 3 SD 3% of the cells (Fig. 4J). There were no statistically significant differences in expression of Caspase 3 for any of the transport simulation groups compared to the control (Fig. 4J).

In summary, every LEC sheet in both control and groups that were subjected to transport simulation, contained cells of varying phenotype, from putative stem cells to terminally differentiated corneal epithelial cells, but with a high ratio of immature and cycling cells. The LEC sheets resembled each other in terms of phenotype variations, and no significant changes in the immunohistochemical markers were observed between groups.

### Effect of Transportation on Metabolic Status of the Medium

**Figure 5:**
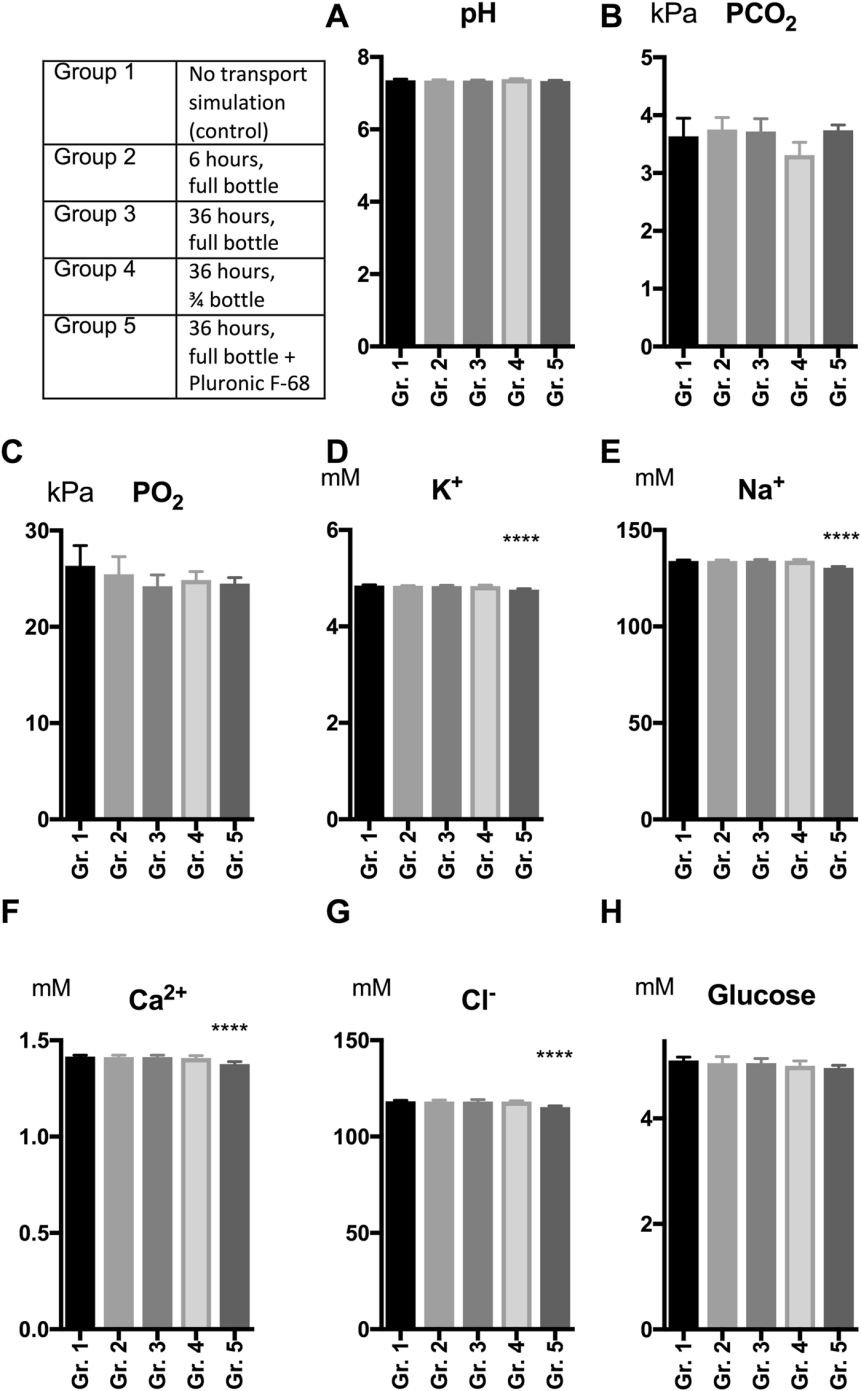
Metabolic Changes in the Storage/Transport Medium. Bar charts of the mean values of metabolic data from samples of medium after storage and/or transport in the Transport Simulation Study. Error bars = 1 Standard Deviation. ****p < 0.0001 compared to the control. **A** = pH, **B** = pCO_2_, **C** = pO_2_, **D** = K^+^, **E** = Na^+^, **F** = Ca^2+^, **G** = Cl^−^, **H** = Glucose.

The capacity of the HEPES-buffered MEM medium to sustain atmospheric storage and transport-simulation was investigated with gas, electrolyte and glucose level measurements from the containers. Bar charts of comparisons between the experimental groups for all metabolic data and are presented in Fig. 5 and the full dataset on metabolic data in the study is available as Supplementary Data S5.

Mean pH for non-transported samples were 7.36 SD 0.03 (Fig. 5A). There were no statistically significant differences in mean pH for any of the transport simulation groups compared to the non-transported control (Fig. 5A). All pH values measured were within optimal ranges for cell growth (7.0–7.4)^25^ and in the lower range of the physiologic level (7.38 +/− 0.02)^26^.

Mean pCO_2_ for non-transported samples were 3.64 SD 0.31 kPa (Fig. 5B). No statistically significant changes in mean pCO_2_ were found for any of the transport simulation groups (Fig. 5B). Mean pCO_2_ for both non-transported and transport simulated samples were below the normal physiologic range (4.6–6.0 kPa)^26^.

Mean pO_2_ for non-transported samples were 26.3 SD 2.1 kPa (Fig. 5C). There were no statistically significant differences in mean pO_2_ for any of the transport simulation groups compared to the non-transported control (Fig. 5C). All pO_2_ measurements were above the normal physiologic range (10.0–14.0 kPa)^26^.

Mean K^+^ for non-transported samples were 4.85 SD 0.02 mmol/L (Fig. 5D). All K^+^ levels measured were within the range of physiologic serum-K^+^ levels (3.5–5.0 mmol/L)^26^. Mean K^+^ concentration was significantly reduced to 4.76 SD 0.02 mmol/L (p < 0.0001) in the 36-hour transport simulation group with containers full of medium supplemented with Pluronic F-68, compared to the non-transported samples (Fig. 5D).

Mean Na^+^ for non-transported samples were 134 SD 0.6 mmol/L (Fig. 5E). All Na^+^ levels measured were below the physiologic range for serum-Na^+^ (135–145 mmol/L)^26^. Mean Na^+^ concentration was significantly reduced to 130 SD 0.6 (p < 0.0001) in the 36-hour transport simulation group with containers full of medium supplemented with Pluronic F-68, compared to the non-transported samples (Fig. 5E).

Mean Ca^2+^ for non-transported samples were 1.4 SD 0.01 mmol/L (Fig. 5F). All Ca^2+^ levels measured were below the range for physiologic serum-Ca^2+^ (2.0–2.6 mmol/L)^26^. Mean Ca^2+^ concentration was significantly reduced to 1.40 SD 0.01 mmol/L for the 36-hour transport simulation group with containers full of medium supplemented with Pluronic F-68, compared to the non-transported group (p < 0.0001, Fig. 5F).

Mean Cl^−^ for non-transported samples were 118 SD 0.5 mmol/L (Fig. 5G). All Cl^−^ levels measured were above the physiologic range for serum-Cl^−^ (95–105 mmol/L)^26^. Mean Cl^−^ concentration was significantly reduced to 115 SD 0.2 (p < 0.0001) for the 36-hour transport simulation group with containers full of medium added Pluronic F-68, compared to the non-transported samples (Fig. 5G).

The mean level of glucose in the media was for the non-transported group 5 SD 0.05 mmol/L (Fig. 5H). The glucose levels did not vary significantly for any of the transport conditions compared to the control (Fig. 5H), and was within the recommended physiologic range of glucose serum-levels (4.0–6.0 mmol/L)^26^.

In summary, the HEPES-MEM based medium was sustainable to atmospheric storage for 4 days including transport simulation for up to 36 hours in terms of pH, pCO_2,_ pO_2_ and glucose levels. All K^+^ levels measured were within the range of physiologic Serum-K^+^ levels. There were minor differences from normal serum-levels for the other electrolytes measured (Na^+^, Cl^−^ and Ca^2+^).

## Discussion

In the present study, we found that the simulation of transport under a variety of conditions appeared not to be critical for HLEC sheets during a storage time of 4 days.

Previously, in Utheim *et al*.^5^ our research group demonstrated that HEPES-MEM storage of LECs at 23 °C maintained cell viability, phenotype and ultrastructure for 4 and 7 days compared to a non-stored control. In the Utheim *et al*. study from 2015 the conditions were identical to the present study in terms of culture method, culture/storage/transport scaffold, storage time, storage medium, and storage temperature^5^. Also, the system with hanging the polyester inserts from the lid of the bottle, freely suspended in a large volume of medium in a sealed container were similar between the present and previous study^5^. In Utheim *et al*. (2015), 4 storage conditions were compared to an unstored, cultured HLEC control group^5^. The storage conditions were 1) storage for 4 days in Quantum 286, 2) storage for 4 days in HEPES-MEM, 3) storage for 7 days in Quantum 286, and 4) storage for 7 days in HEPES-MEM. In the present study, HLEC sheets stored in HEPES-MEM for 4 days are used as controls, that is, consistent with experimental group no 2) in the Utheim *et al*. (2015) study, which was found to be unchanged compared to the non-stored control group of cultured LECs^5^. In Utheim *et al*. (2015) we found that viability for the unstored (n = 10) cultures were 98 SD 3%, which was not statistically significant different from HEPES-MEM stored for 4 days (n = 10, viability 97 SD 1%, p = 0.43). Furthermore, in Utheim *et al*. (2015) HE sections showed multi-layered, intact HLEC sheets both for un-stored (n = 7) and HEPES-MEM stored groups (n = 5 for HEPES-MEM 4 days). Finally, phenotype (markers ∆Np63α, p63, Bmi-1, C/EBP∂, ABCG2 and K19 for immature cells, K3 and Cx43 for differentiated cells), was predominantly immature, with no statistically significant differences for any of the immunohistochemical markers for the HEPES-MEM 4 days of storage group compared to the non-stored control (n = 6–7 for HEPES-MEM 4 days of storage and n = 6–7 for non-stored control)^5^. Because of these results we used stored tissues as controls in the present study. Moreover, other studies of epithelial sheets on HAM using similar storage medium^27^, storage methods, storage temperature and storage time^13,14,28,29^ have demonstrated good preservation of the tissue compared to non-stored controls. Consequently, the results of the present study in light of the Utheim *et al*. (2015) study^5^ and the above mentioned previous storage studies, suggest that combined transport and storage with the present method can be performed without damage to the cultured LEC sheets.

The viability was high in all LECs in the present study independent of transport condition (97 SD 3.1% viable cells for non-transported, no significant differences for any transport simulation group), presented as live cells in percentage of total number of cells in basal layer epithelia micrographs after exposure to calcein/ethidium homodimer. This finding correspond with the previous study of HEPES-MEM storage of HLECs, where mean viability for non-stored cultured HLECs were 98 SD 3% using the same analysis, without significant differences for HLECs stored for 4 and 7 days^5^. As regards to other studies of transportation of ocular surface epithelial sheets, Oie *et al*. found a viability of 72% before and 76.3% after a 5 hour long airplane transportation of LEC and oral keratinocytes, with a dye exclusion test of a cell suspension subjected to flow cytometry^17^. Vasania *et al*. found a viability of 78–90% after transport of up to 48 hours for conjunctival tissue^16^. The present study suggests that vigorous shaking does not appear to cause cell death.

In the present study the epithelial sheets remained intact and attached to the HAM regardless of transport situation. In previous HLEC storage studies on HAM, the epithelial sheets fell apart after a prolonged storage time of 3 weeks^15^ of ambient temperature. They also failed after 7 days in 5 °C Optisol GS-storage and 31 °C organ culture storage^13^, but remained intact for 7 days of storage in both serum-containing^13,14^ and serum-free^5^ medium at 23 °C. Also, Wright *et al*. found 18–22 °C to be superior to 4 °C and 37 °C for preservation of human keratinocytes and rabbit limbal epithelial cells by the use of an biomimetic alginate gel as substrate^12^. In the same study, these authors found that viability was good after 7 days of storage, but the cells did non remain alive for longer periods of time. Consistent with the results of Wright *et al*.^12^ and our above mentioned previous studies, we suggest that temperature and time may be more critical to the preservation of an intact epithelial sheet than shaking during transport.

Despite the finding that the HLEC sheets remained well attached to the HAM, the numbers of hemidesomsomes and desmosomes decreased in the experimental group with containers partially filled with medium, compared to the stored, but non-transported control. Oie *et al*. also found a reduction in tight junctions in cultured oral mucosal epithelium and LEC sheets after transport, assessed by expression of the immunohistochemical marker ZO-1^17^. Thus, an air liquid interface during transport could reduce the mechanical strength of the tissue, suggesting that an air-liquid interface in transport containers should be avoided.

In the present study a phenotype of predominantly immature cells was demonstrated, that was similar to previously reported results using non-stored cultured HLEC^5^. The present study did not reveal any alterations in the phenotype of LEC for any of the storage/transport conditions.as found in transportation studies on conjunctival epithelial cells^16^, corneal endothelial cells^30^; and rabbit LEC/human oral mucosal epithelial cells^17^.

The purpose of transplanting the cultured epithelial sheets is to restore the stem cell population in eyes with LCSD. Hence, it is crucial that storage and transport conditions do not promote differentiation of cells. In particular, the expression of transcription factor p63 and its isotype ΔNp63α is of interest. In 2010, Rama *et al*. found that a successful post-operative outcome following LEC transplantation was associated with more than 3% of the cells staining brightly for 4A4 anti-p63^31^. In addition, ΔNp63α is known to be the most abundant isotype of p63 in limbal tissue^19^, and to mark holoclones^18^. we We found both moderate cytoplasmic staining of ΔNp63α and strong nuclear staining of p63 and infrequently ΔNp63α in our study. Our results cannot be directly compared with the work of Rama *et al*.^31^ due to differences in methods (automated quantitative-fluorescence-immunohistochemistry versus conventional immunohistochemistry analysis in the present study). Nevertheless, in the present study transportation itself did not cause any depletion of p63 and/or ΔNp63α positive cells.

Several aspects within the transport container may have contributed to successful preservation despite a prolonged transportation simulation of 36 hours. First, the epithelial sheets were not removed from the inserts, hence providing mechanical strength to the epithelial cell sheet during transport. Second, the inserts were freely suspended from the rubber caps of the containers, which would redirect and absorb shear forces during transport. Third, the large amount of transport/storage medium is likely to dilute toxic metabolites and provide nutriments without change in medium needed.

Interestingly, lack of external oxygen for the four days of storage and transport simulation did not reduce the level of pO_2_ in the medium. This may be due to a favourable combination of the use of large containers with abundant amounts of medium and low metabolism of LEC sheets, which is reflected in the high and stable glucose levels of the medium. Our results are consistent with those of Oie *et al*. who also described successful transport with no external oxygen source for 12 hours at temperatures of 32–35 °C.

We found that the additive Pluronic F-68 reduced the electrolyte-levels of the medium. The changes in electrolyte levels were not shown to have any impact on viability, phenotype or ultrastructure of the transported cells. Thus, the study did not reveal clear beneficial effects of Pluronic F-68 in preserving LEC sheets, and we therefore suggest that Pluronic F-68 as an additive should be avoided.

In the present study, we showed that cultured epithelial cells sheets appear to endure 36 hours of vigorous shaking under laboratory conditions that mimics transportations. However, there are other effects that the current transport simulations do not test. For example, we did not simulate the acceleration/deceleration that occurs during take-off and landing of an aircraft, or other movements that might influence the epithelial sheets. These aspects of transport remain to be tested either in the laboratory or in an actual transportation situation.

## Conclusions

In conclusion, cultured LEC sheets appear to endure vigorous shaking for at least 36 hours if the container is full. These findings suggest that transportation for 36 hours is possible, which will cover most areas in the world, connecting highly specialized labs to eye clinics in need of transplants.

## Methods

### Harvesting of Limbal Donors and Ethics Statement

Limbal rings from three cadaveric donors were harvested at Barraquer Ophthalmology Centre in Spain on day two, four and six post mortem, and shipped to Oslo University Hospital in Norway. Time from harvest to culture was three, six and seven days while time from death to culture was 10 to 11 days.

The research was conducted in accordance with the Declaration of Helsinki. Written, informed consent from the next of kin was obtained by personnel at the Eye Bank of Barraquer Ophthalmology Centre for the use of limbal donor tissue for research purposes. The appropriate authorities approved all the transfers of the limbal tissue from Spain to Norway. HAM were donated after written, informed consent from healthy women who had undergone elective caesarean section at Oslo University Hospital, Norway. The Norwegian Regional Committee for Medical and Health Research Ethics approved the collection and banking of HAM and the use of ocular tissue.

### Preparation and Culture of Limbal Epithelial Cells

Prior to the experiments, the limbal rings were prepared and cultured for 14 days on HAM, as previously described by Meller *et al*.^32^. In brief, HAM preserved as previously reported^33^ were thawed, rinsed and sutured to the polyester membrane of culture plate inserts. Limbal rings were divided in approximately 3 × 3 mm explants, exposed to dispase for five minutes, and placed on the inserts with the epithelial side facing the intact HAM, one explant per insert.

The explants were incubated for 14 days at 37 °C with 5% CO_2_ in the culture medium, which consisted of HEPES-buffered DMEM with sodium-bicarbonate and Ham’s F12. This culture medium was supplemented with 5% foetal bovine serum, 0.5% dimethyl sulphoxide, 2 ng/mL human epidermal growth factor, 5 µg/mL insulin, 5 µg/mL transferrin, 5 ng/mL selenium, 3 ng/mL hydrocortisone, 30 ng/mL cholera toxin, 50 µg/mL gentamycin, and 1.25 µg/mL amphotericin B. The culture medium was changed every three days.

### Storage and Transport Simulation

After culture, each polyester membrane was separated from the insert with a surgical blade, sutured loosely to the rubber caps of glass infusion-containers in order to let the culture hang freely from the cap on the inside, (Supplementary Fig. S3), and submerged in xenobiotic-free MEM with Earle’s salts and L-glutamine (used due to its beneficial preserving properties)^5^. The medium was buffered with 25 mM HEPES and 22 mM sodium bicarbonate and added 50 *μ*g/ml gentamicin, 100 *μ*g/ml vancomycin and 2.5 *μ*g/ml amphotericin B.

All cultured LEC sheets were subjected to storage at ambient temperature for 1 to 3 hours prior to transport simulation, and from 2.5 to 4 days after transport simulation so that the total length of storage and transportation time was 4 days for all groups. Explants from the three cadaveric donors were equally spread between the transport simulation groups. The LEC sheets serving as controls (group 1) were stored under the same conditions as the LEC sheets in the experimental groups, but did not undergo transport simulation. For the other groups, the LEC sheets were shaken at 200 orbital rotations per minute (rpm) on an Edmund Bühler KL-2 Multi-purpose mixer shaker (POCD Scientific, Hamburg, Germany). Group 2-sheets were subjected to 6 hours’ shaking, while group 3–5 underwent 36 hours’ shaking before storage. Group 4-sheets were transported and stored in containers three quarters filled with medium (47 mL), while containers filled to the cap with medium (63 mL) were used for the other groups. In the fifth group, the medium was added the surfactant agent Pluronic F-68 in 0.3% solution. We did not use non-stored controls as our previous experiments demonstrated that the viability and phenotype of LECs under non-stored compared to stored conditions was not different.

At the end of the experiments, each LEC sheet was divided into three pieces, for viability analysis, TEM and histochemical/immunohistochemical analysis. Metabolic analyses were performed on samples of the storage medium. The results from the four transport simulations were compared with the non-transported controls.

### Cell Viability Analysis

Cell viability analysis was performed using a calcein-acetoxymethyl ester (CAM)/ethidium homodimer 1 (EH-1) assay (Invitrogen, Oslo, Norway) as described elsewhere^34^, with some modifications. In brief, LEC sheets were incubated in phosphate-buffered saline (PBS) containing 2 mM CAM and 2 mM EH-1 (23 °C for 45 min, protected from light), then washed with PBS, and subsequently mounted on cover-slipped glass slides. Fluorescent images of the basal layer were captured using an Axiovert 100 LSM 510 laser scanning confocal microscope (Carl Zeiss Microscopy, Oberkochen, Germany). The numbers of live (CAM-stained; green fluorescence) and dead (EH-1 stained; red fluorescence) cells were counted in five fields per sample at a magnification of 250× by two independent investigators blinded to the origin of the samples. The percentage of viable cells per LEC sheet was calculated as live cells/(live cells + dead cells) × 100%. Three-week LEC cultures (n = 2) exposed to methanol for 1 hour were used as positive controls for dead cells.

### TEM Analysis

The cultured LEC sheets were processed for TEM analysis as previously described^13^. In brief, the tissues were fixed in 2% glutaraldehyde in 0.2 M cacodylate buffer adjusted to pH 7.4, post-fixed in 1% osmium tetroxide, and dehydrated through a graded series of ethanol up to 100%. The tissue blocks were immersed in propylene oxide twice for 20 minutes and embedded in Epon. Ultrathin sections were cut on a microtome (Ultracut Ultramicrotome UCT; Leica, Wetzlar, Germany) and examined with a transmission electron microscope (model CM120; Philips, Eindhoven, The Netherlands). Images were captured at 9700× magnification of both basal and suprabasal layers from three pre-defined positions (the two edges and centre of each sample) by an experienced technician. An investigator counted the number of desmosomes in the supra-basal layers and the number of hemi-desmosomes in the basal layers of the micrographs. The numbers were presented per 100 µm of epithelium. In addition, full-thickness images of the epithelia were taken at 3400× magnification and the numbers of epithelial cell layers were counted. The technician and the investigator were blinded to the origin of the samples.

### Histological Analysis

Serial sections of 3.5 *μ*m were mounted on SuperFrost®Plus slides and stained with H&E. Sections from transported and non-transported LEC sheets (n = 28) were photographed and examined with a light microscope camera at a magnification of 400×, with respect to shedding of cells, epithelial attachment to the HAM, and stratification of the epithelia. Thickness of the cultured LEC sheets (HAM not included) was measured in eight pre-defined positions within the outgrowth area of the LEC sheets; 250 *μ*m, 500 *μ*m, 750 *μ*m, and 1000 *μ*m away from the limbal donor piece in two opposite directions.

### Immunohistochemical Analyses

Serial sections of 3.5 *μ*m were mounted on SuperFrost®Plus slides, incubated with antibodies at 37 °C overnight using Benchmark XT Antibody diluent (251-018). Thereafter, the detection kit Ventana ultraView Universal DAB (760-500) was used; an automated immunostaining system based on the ABC avidin-biotin-peroxidase method, with negative and positive controls (Ventana Medical Systems Inc. Tucson, AZ, USA). The immunohistochemical markers used in the study are presented in Table 1.

**Table 1:** Antibodies Used in the Study.

Antigen	Dilution	Clone	Company
p63	1:25	p63 protein mouse monoclonal clone 4A4. Code No M72747	DAKO Cytomation Norden A/S, Glostrup, Denmark
ΔNp63α	1:200	ΔNp63α antibody. Rabbit, polyclonal, custom made.	Primm, Milano, Italy
C/EBPδ	1:100	C/EBPδ antibody. Rabbit, polyclonal, ab65081	Abcam, Cambridge, MA, USA
Bmi1	1:20	Bmi1 antibody. Rabbit, polyclonal, ab97729	Abcam
ABCG2	1:20	ABCG2 protein. Mouse, monoclonal clone bxp-21.	Sigma-Aldrich, St Louis, MO, USA
K3	1:500	K3, Clon AE5 Mouse anti-cytokeratin	ImmuQuest, Cleveland, UK
Ki67	1:75	Ki67 Mouse monoclonal MIB-1	DAKO
PCNA	1:3500	PCNA. Mouse monoclonal, M879	DAKO
Caspase-3	1:200	Cleaved Caspase-3, Asp175, 5A1E, Rabbit monoclonal antibody	Cell Signaling, Danvers, MA, USA

One experienced investigator (counter 1) counted negative and positively stained cells for all markers, through a light microscope at 400× magnification based on a pre-set list of criteria. An independent investigator (counter 2) counted negative and positively stained cells for some of the markers, and the results between counter 1 and counter 2 were averaged. The expression of the various markers per LEC sheet was calculated as follows: (the number of positive cells/total number of cells) × 100%. In addition, regional variations within the epithelia were assessed in basal versus supra-basal epithelial layers after a previously described grading system^35^, (Supplementary Table S1).

### Metabolic Analyses

200 *μ*L of HEPES-MEM based medium from each storage container was sampled with a syringe after termination of the experiment and analysed directly on a Rapid point 405 blood gas analyzer (Siemens Healthcare, Erlangen, Germany) for pH, pCO_2_, pO_2_, Na^+^, K^+^, Ca^2+^, Cl^−^, and glucose.

### Statistical Analyses

One-way analysis of variance (ANOVA) was used to compare all the transport simulation groups with the control group. To adjust for multiple comparisons, Tukey’s post hoc test was used if equal variances were verified by Levene’s test of homogeneity of variances. The Dunnett’s T3 post hoc test was applied if equal variances were not verified by Levene’s test. If data sets were not normally distributed, non-parametric tests were performed in addition, but did not alter the conclusions of the hypothesis testing. The results from the five experimental groups are presented as mean SD 1× standard deviation (SD). A significance level of 5% was used throughout the study. SPSS version 21.0; SPSS Inc., Chicago, USA and GraphPad Prism version 7 were used for the statistical analyses and creating of graphs.

### Image processing

Adobe Photoshop version 12.1 is used to adjust brightness and contrast. This is applied equally across images and equally for all experimental groups.

### Data Availability

All data generated or analysed during this study are included in this published article and its Supplementary Information File.

## Electronic supplementary material

Supplementary Dataset 1
