# Supplementary material for: Effect of Transportation on Cultured Limbal Epithelial Sheets for Worldwide Treatment of Limbal Stem Cell Deficiency
Source: Sci Rep. 2018 Jul 12;8:10502. doi: 10.1038/s41598-018-28553-0 (PMC6043629; doi:10.1038/s41598-018-28553-0)
Supplement: Supplementary file 1 — Supplementary Dataset 1 [file 41598_2018_28553_MOESM1_ESM.pdf]

Supplementary Data File

Effect of Transportation on Cultured Limbal Epithelial Sheets for Worldwide Treatment of Limbal Stem Cell Deficiency

O.A. Utheim[1, 2], T. Lyberg[1], J.R. Eidet[1,2]*, S. Raeder[3], A. Sehic[4], B. Roald[5], E. Messelt[4], M. F. de la Paz[6], D. A. Dartt[7], and T.P. Utheim[1,2,3,4]*

[1]Department of Medical Biochemistry, Oslo University Hospital, Oslo, Norway; [2]Department of Ophthalmology, Oslo University Hospital, Oslo, Norway; [3] Norwegian Dry Eye Clinic, Oslo, Norway; [4]Department of Oral Biology, Faculty of Dentistry, University of Oslo, Oslo, Norway; [5]Department of Pathology, Oslo University Hospital, Oslo, Norway; [6]Institut Universitari Barraquer, Universitat Autonoma de Barcelona, Barcelona, Spain; [7]Schepens Eye Research Institute / Massachusetts Eye and Ear Infirmary, Department of Ophthalmology, Harvard Medical School, Boston, MA, USA.

**Shared second authorship*

Content:

Supplementary Table S1	page 3
Supplementary Figure S1	page 4
Supplementary Figure S2	page 5
Supplementary Figure S3	page 6
Supplementary DataS1	page 7 – 10
Supplementary Data S2	page 11 – 12
Supplementary Data S3	page 13
Supplementary Data S4	page 14 – 22
Supplementary Data S5	page 23 – 24

Supplementary Table S1. Regional Variations in Immunohistochemical Expression for Cultured LEC in the Transport Simulation Study.

		No transport	36 hours, full bottle	6 hours, full bottle	36 hours, ¾ bottle	36 hours, full bottle + Pluronic F-68
p63	sb	(+)	(+)	(+)	(+)	(+)
	b	+	+	+	+	+
∆NP63α	sb	+++	+++	+++	+++	+++
	b	++++	++++	++++	++++	++++
C/EBP∂	sb	+++(+)	+++	+++	+++	+++
	b	++++	++++	++++	++++	++++
Bmi-1	sb	+	0	0	0	0
	b	+	+	+	+	+
ABCG2	sb	++++	++++	++++	+++	++++
	b	++++	++++	++++	+++(+)	++++
K3	sb	+++(+)	+++	+++(+)	+++(+)	+++(+)
	b	++++	++++	++++	++++	++++
Ki67	sb	+(+)	+(+)	++	++(+)	++(+)
	b	+	+	+	++	++
PCNA	sb	+++(+)	+++	+++(+)	+++	+++
	b	++++	++++	++++	++++	++++
Caspase-3	sb	(+)	(+)	(+)	+	(+)
	b	(+)	(+)	(+)	+	(+)

Variations in immunohistochemical expression between basal (b) and suprabasal (sb) layers of the limbal epithelial cell (LEC) sheets, based on semi-quantitative assessment of sections.
0 = not detectable, (+) = mostly detectable in < ¼ of cells, + = detectable in < ¼ of cells, +(+) = mostly detectable in ¼ - ½ of cells, ++ = detectable in ¼ - ½ of cells, +++(+) = mostly detectable in > ¾ of cells, +++ = detectable in ½ - ¾ of cells, ++++ = detectable in > ¾ of cells.

Supplementary Figure S1. Mean Morphology Data of Limbal Epithelial Cells (LEC) Sheets in the Transport Simulation Study.

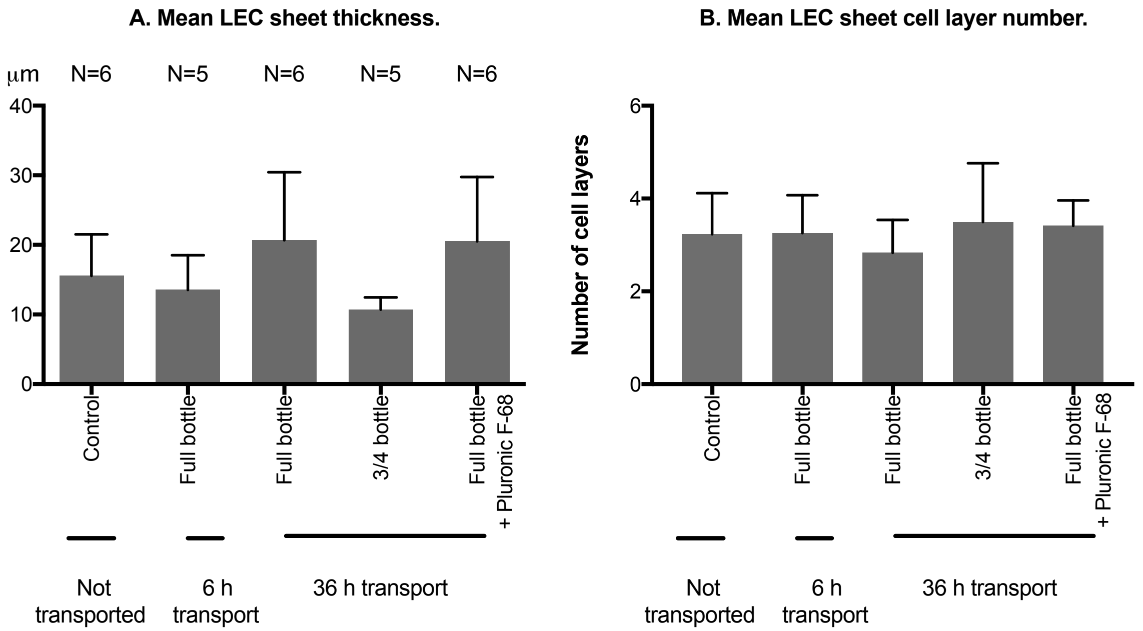

Average values of A) central thickness (in μm) and B) number of cell layers for limbal epithelial cell (LEC) sheets for four different transport simulation conditions versus a control group. Central thicknesses (A) are measured from micrographs of H&E sections. Number of cell layers (B) are calculated from Transmission Electron Microscopy micrographs. There were not any significant changes in A) thickness and B) cell layer number for any of the transport simulation groups compared to the control. Groups are 1) Stored, but not transported control 2) 6 hours' transport simulation with bottles full of medium 3) 36 hours' transport simulation with bottles full of medium 4) 36 hours' transport simulation with bottles three quarters full of medium 5) 36 hours' transport simulation with bottles full of medium added the shear protecting agent Pluronic F-68. Error bar = 1 standard deviation.

Supplementary Figure S2. Staining pattern of Immunohistochemical Markers.

Staining of Immunohistochemical Markers from representative areas of Cultured Limbal Epithelial Cell Sheets. The following markers were used: **A)** pan-p63, **B)** ΔNP63α, **C)** C/EBP∂, **D)** Bmi-1, **E)** ABCG2, **F)** Keratin 3, **G)** Ki67, **H)** PCNA, **I)** Caspase-3. I) represents stored, but not-transported cultures, II) transport simulation for 6 hours in full bottle, III) transport simulation for 36 hours in full bottle, IV) transportation simulation for 36 hours in bottle three quarters filled with medium, V) transportation simulation for 36 hours in full bottle with medium added the surfactant Pluronic-F68. Original magnification 400x.

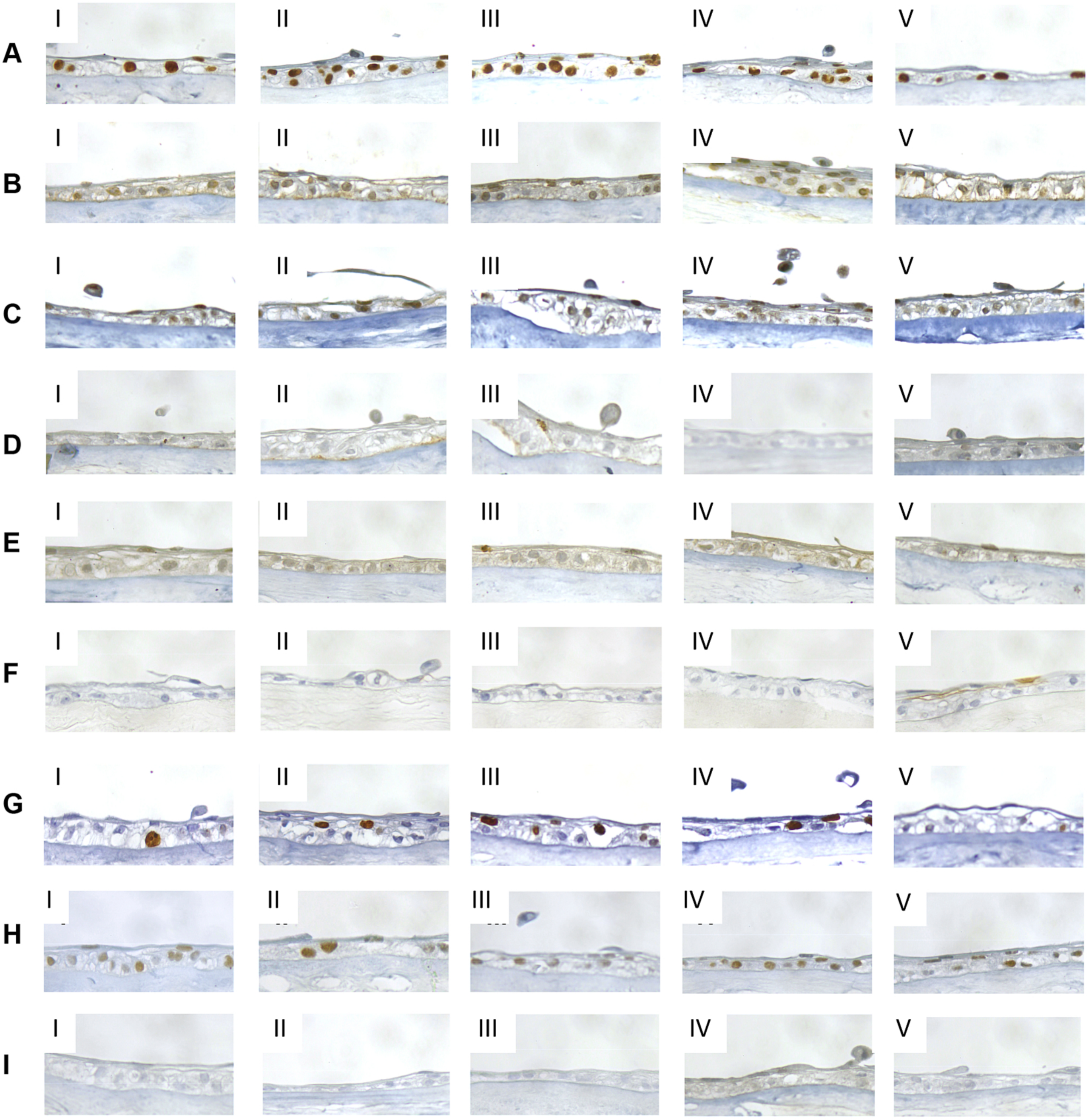

Supplementary Figure S3. Illustration of the principals behind storage and transport of cultured limbal epithelial cells (LEC) for the treatment of limbal stem cell deficiency (LSCD).

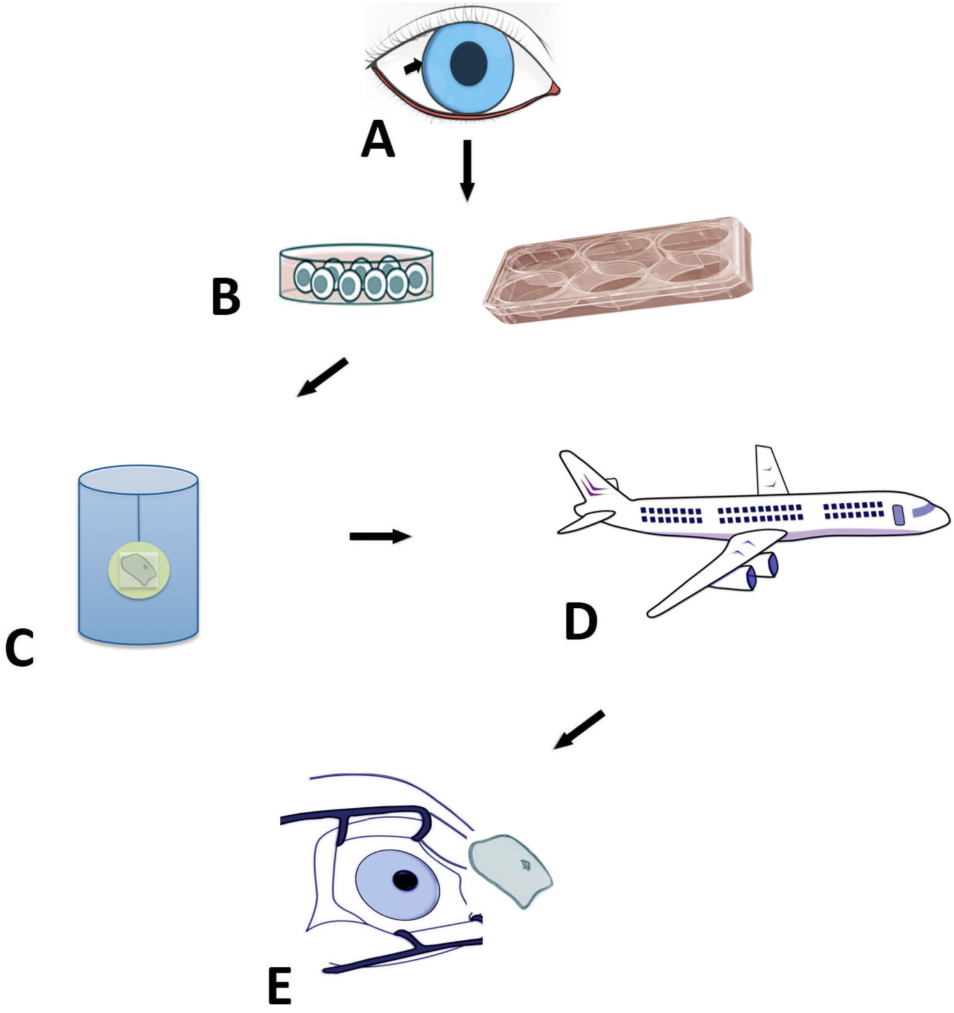

Harvesting of limbal epithelial cells (LEC)s from a healthy donor eye (**A**), culture and expansion of LECs **(B)** before enclosing the cultured LEC sheets in containers as described in the study **(C),** enables worldwide transportation **(D)** and increased availability and safety of LSCD treatment with LEC transplantation **(E)**.

Supplementary Dataset S1.
Viability data underlying the analyses in the study.

Group 1 = Non-transported control group.
Group 2 = transport simulation for 6 hours in full container.
Group 3 = Transport simulation for 36 hours in full container.
Group 4 = Transport simulation for 36 hours in 3/4 filled container.
Group 5 = Transport simulation for 36 hours in full container added Pluronic F-68.

Group	Red	Green	Total cells	% Viable cells per image	Number of cultured HLEC sheets (5 images per sheet)
1	3	46	49	94	
1	7	109	116	94	
1	10	20	30	67	
1	5	44	49	90	
1	9	152	161	94	
1	15	284	299	95	
1	11	311	322	97	
1	13	167	180	93	
1	10	147	157	94	
1	11	105	116	91	
1	3	70	73	96	
1	2	23	25	92	
1	1	84	85	99	
1	1	148	149	99	
1	0	76	76	100	
1	4	218	222	98	
1	9	246	255	96	
1	9	171	180	95	
1	9	194	203	96	
1	5	234	239	98	
1	1	98	99	99	
1	0	176	176	100	
1	2	202	204	99	
1	0	93	93	100	
1	0	106	106	100	
1	2	207	209	99	

1	1	147	148	99	
1	5	108	113	96	
1	1	293	294	100	
1	0	204	204	100	6
2	3	92	95	97	
2	3	95	98	97	
2	0	0	0	0	
2	2	109	111	98	
2	2	83	85	98	
2	8	254	262	97	
2	9	263	272	97	
2	11	257	268	96	
2	5	124	129	96	
2	7	209	216	97	
2	0	160	160	100	
2	1	178	179	99	
2	1	238	239	100	
2	2	270	272	99	
2	3	180	183	98	
2	3	242	245	99	
2	8	217	225	96	
2	12	189	201	94	
2	7	210	217	97	
2	3	271	274	99	
2	6	267	273	98	
2	1	115	116	99	
2	0	70	70	100	
2	1	80	81	99	
2	3	53	56	95	
2	7	90	97	93	
2	7	45	52	87	
2	6	62	68	91	
2	8	74	82	90	
2	1	94	95	99	
2	5	50	55	91	6
3	5	74	79	94	
3	10	11	21	52	
3	6	135	141	96	
3	6	88	94	94	
3	10	140	150	93	
3	6	129	135	96	
3	6	52	58	90	
3	6	79	85	93	

3	8	146	154	95	
3	2	173	175	99	
3	0	273	273	100	
3	4	279	283	99	
3	3	294	297	99	
3	2	196	198	99	
3	2	308	310	99	
3	1	273	274	100	
3	1	327	328	100	
3	3	234	237	99	
3	6	114	120	95	
3	1	169	170	99	
3	1	255	256	100	
3	3	124	127	98	
3	1	76	77	99	
3	0	135	135	100	
3	3	243	246	99	
3	2	196	198	99	
3	7	101	108	94	
3	4	167	171	98	
3	6	117	123	95	
3	10	167	177	94	6
4	3	205	208	99	
4	7	291	298	98	
4	5	238	243	98	
4	8	247	255	97	
4	4	252	256	98	
4	2	51	53	96	
4	11	31	42	74	
4	3	24	27	89	
4	4	92	96	96	
4	8	33	41	80	
4	0	109	109	100	
4	0	71	71	100	
4	0	198	198	100	
4	0	246	246	100	
4	4	148	152	97	
4	6	85	91	93	
4	7	147	154	95	
4	6	54	60	90	
4	2	149	151	99	
4	3	55	58	95	
4	3	169	172	98	

4	2	132	134	99	
4	2	189	191	99	
4	0	198	198	100	
4	1	256	257	100	
4	0	69	69	100	
4	1	109	110	99	
4	2	80	82	98	
4	6	75	81	93	
4	1	74	75	99	6
5	4	52	56	93	
5	2	72	74	97	
5	6	42	48	88	
5	3	32	35	91	
5	9	121	130	93	
5	2	348	350	99	
5	1	97	98	99	
5	2	101	103	98	
5	1	167	168	99	
5	7	170	177	96	
5	1	340	341	100	
5	1	301	302	100	
5	1	261	262	100	
5	0	297	297	100	
5	2	225	227	99	
5	2	190	192	99	
5	4	220	224	98	
5	3	202	205	99	
5	2	121	123	98	
5	6	165	171	96	
5	1	206	207	100	
5	0	180	180	100	
5	4	203	207	98	
5	0	144	144	100	
5	2	207	209	99	
5	9	108	117	92	
5	12	153	165	93	
5	3	216	219	99	
5	1	217	218	100	
5	9	226	235	96	6

Supplementary Dataset S2.
Datasets underlying the analyses of cultured limbal epithelial cell (LEC) sheet thickness in the study.

A

In μm, measured on H&E micrographs

Group	Average per LEC sheet
1	16.1
1	20.4
1	24.1
1	9.9
1	8.6
1	14.3
2	7.9
2	15.9
2	14.5
2	x
2	20.1
2	9.2
3	8.0
3	22.1
3	11.5
3	34.9
3	22.0
3	25.5
4	11.4
4	11.2
4	7.8
4	10.5
4	x
4	12.5
5	14.7
5	15.4
5	20.9
5	33.3
5	9.3
5	29.4

B

In number of cell layers, measured on TEM micrographs

Group	Average per LEC sheet
1	3.66
1	3.83
1	3.5
1	3.5
1	1.66
2	3.33
2	3.66
2	4
2	2.83
2	1.83
2	3.88
3	2.83
3	3.5
3	3.5
3	2.5
3	1.83
4	2.5
4	6
4	3.33
4	3
4	2.83
4	3.33
5	3.16
5	4
5	3.83
5	3.83
5	3
5	2.66

X= Missing data. Group 1 = Non-transported control group.
Group 2 = transport simulation for 6 hours in full container.
Group 3 = Transport simulation for 36 hours in full container.
Group 4 = Transport simulation for 36 hours in 3/4 filled container.
Group 5 = Transport simulation for 36 hours in full container added Pluronic F-68.

Supplementary Dataset S3.
The numbers of desmosomes and hemi-desmosomes per culture underlying the desmosome/hemi-desmosome analyses in the study.

Calculated from Transmission Electron Microscopy micrographs .
X= Missing data. Group 1 = Non-transported control group.
Group 2 = transport simulation for 6 hours in full container.
Group 3 = Transport simulation for 36 hours in full container.
Group 4 = Transport simulation for 36 hours in 3/4 filled container.
Group 5 = Transport simulation for 36 hours in full container added Pluronic F-68.

Group	Desmosomes / 100 µm	Hemi-desmosomes / 100 µm
1	23.5	39.9
1	37.6	39.9
1	39.9	44.6
1	30.5	35.2
1	35.2	32.9
2	58.7	23.5
2	46.9	32.9
2	65.7	39.9
2	23.5	x
2	46.9	32.9
2	32.9	39.9
3	32.9	23.5
3	58.7	61.0
3	51.6	28.2
3	39.9	54.0
3	35.2	32.9
4	16.4	7.0
4	18.8	14.1
4	14.1	16.4
4	4.7	21.1
4	7.0	21.1
4	14.1	7.0
5	79.8	35.2
5	89.2	70.4
5	46.9	30.5
5	56.3	35.2
5	x	39.9
5	32.9	23.5

Supplementary dataset S4. Positive versus negatively stained cells per culture for immunohistochemical markers p63, ΔNp63α, C/EBPδ, Bmi1, ABCG2, K3, Ki67, PCNA, and Caspase 3.

p63

Group 1	Positive/negative/total number of cells counted	Percentage of positive cells	Percentage of negative cells
	90/10/100	90	10
	98/2/100	98	2
	99/1/100	99	1
	94/6/100	95	6
	81/19/100	81	19
	94/6/100	94	6
Group 2	98/2/100	98	2
	94/6/100	94	6
	95/5/100	95	5
	91/9/100	91	9
	x	x	x
Group 3	92/8/100	92	8
	99/1/100	99	1
	89/11/100	89	11
	93/7/100	93	7
	89/11/100	89	11
	90/10/100	90	10
Group 4	94/6/100	94	6
	94/6/100	94	6
	95/5/100	95	5
	89/11/100	89	11
	x	x	x
	83/17/100	83	17
Group 5	93/7/100	93	7
	99/1/100	99	1
	95/5/100	95	5
	93/7/100	93	7
	95/5/100	95	5
	92/8/100	92	8

x= missing data.
Group 1 = stored, but not transported control.
Group 2 = Transport simulation for 6 hours in full container.
Group 3 = Transport simulation for 36 hours in full container.
Group 4 = Transport simulation for 36 hours in 3/4 filled container.
Group 5 = Transport simulation for 36 hours in full container added Pluronic F-68.

ΔNp63α

Group 1	Cell numbers of dense/granular/no staining	Densley nuclear staining (%)	Granular nuclear staining (%)	No nuclear staining (%)
	7/63/23/93	8	68	26
	0/72/28/10	0	72	28
	2/86/12/10	2	86	12
	1/93/6/100	1	93	6
	0/82/18/10	0	82	18
	0/93/7/100	0	93	7
Group 2	7/67/26/10	7	67	26
	3/65/28/96	3	68	29
	3/95/3/101	3	94	3
	0/91/10/10	0	90	10
	0/100/0/10	0	100	0
Group 3	0/86/14/10	0	86	14
	0/90/10/10	0	90	10
	0/97/4/101	0	96	4
	1/63/28/92	1	69	30
	x	x	x	x
	1/92/7/100	1	92	7
Group 4	0/87/6/93	0	94	6
	0/91/10/10	0	90	10
	0/98/2/100	0	98	2
	2/86/5/93	2	93	5
	x	x	x	x
	0/70/30/10	0	70	30
Group 5	4/22/74/10	4	22	74
	0/86/16/10	0	84	16
	0/98/2/100	0	98	2
	x	x	x	x
	0/96/4/100	0	96	4
	0/55/45/10	0	55	45

x= missing data. Group 1 = stored, but not transported control. Group 2 = Transport simulation for 6 hours in full container. Group 3 = Transport simulation for 36 hours in full container. Group 4 = Transport simulation for 36 hours in 3/4 filled container. Group 5 = Transport simulation for 36 hours in full container added Pluronic F-68.

C/EBPδ

	Positive/negative/total number of cells counted	Percentage of positive cells	Percentage of negative cells
Group 1	58/43/101	57	43
	65/35/100	65	35
	96/5/101	95	5
	80/20/100	80	20
	31/24/55	56	44
	50/61/111	45	55
Group 2	81/19/100	81	19
	49/52/101	49	51
	85/19/104	82	18
	45/55/100	45	55
	32/23/55	58	42
Group 3	40/60/100	40	60
	73/29/102	72	28
	89/12/101	88	12
	72/28/100	72	28
	78/22/100	78	22
	52/48/100	52	48
Group 4	15/39/54	28	72
	78/22/100	78	22
	73/27/100	73	27
	70/30/100	70	30
	x	x	x
	45/55/100	45	55
Group 5	33/71/104	32	68
	39/62/101	39	61
	81/19/100	81	19
	86/15/101	85	15
	78/22/100	78	22
	58/42/100	58	42

x= missing data. Group 1 = stored, but not transported control. Group 2 = Transport simulation for 6 hours in full container. Group 3 = Transport simulation for 36 hours in full container. Group 4 = Transport simulation for 36 hours in 3/4 filled container. Group 5 = Transport simulation for 36 hours in full container added Pluronic F-68.

Bmi1

	Positive/negative/total number of cells counted	Percentage of positive cells	Percentage of negative cells
Group 1	0/100/100	0	100
	4/96/100	4	96
	8/92/100	8	92
	0/100/100	0	100
	9/91/100	9	91
	4/96/100	4	96
Group 2	0/100/100	0	100
	6/94/100	6	94
	0/100/100	0	100
	1/99/100	1	99
	1/99/100	1	99
Group 3	0/100/100	0	100
	6/94/100	6	94
	1/99/100	1	99
	8/92/100	8	92
	4/96/100	4	96
	1/99/100	1	99
Group 4	1/99/100	1	99
	0/100/100	0	100
	1/99/100	1	99
	0/100/100	0	100
	x	x	x
	4/96/100	4	96
Group 5	3/97/100	3	97
	1/99/100	1	99
	5/95/100	5	95
	4/96/100	4	96
	5/95/100	5	95
	6/94/100	6	94

x= missing data. Group 1 = stored, but not transported control. Group 2 = Transport simulation for 6 hours in full container. Group 3 = Transport simulation for 36 hours in full container. Group 4 = Transport simulation for 36 hours in 3/4 filled container. Group 5 = Transport simulation for 36 hours in full container added Pluronic F-68.

ABCG2

	Positive/negative/total number of cells counted	Percentage of positive cells	Percentage of negative cells
Group 1	93/7/100	93	7
	96/4/100	96	4
	90/1/91	99	1
	94/6/100	94	6
	x	x	x
	99/1/100	99	1
Group 2	94/6/100	94	6
	100/0/100	100	0
	100/0/100	100	0
	100/0/100	100	0
	x	x	x
Group 3	94/6/100	94	6
	100/0/100	100	0
	99/1/100	99	1
	100/0/100	100	0
	98/2/100	98	2
	100/0/100	100	0
Group 4	98/2/100	98	2
	97/3/100	97	3
	90/10/100	90	10
	100/0/100	100	0
	x	x	x
	97/3/100	97	3
Group 5	99/1/100	99	1
	97/3/100	97	3
	95/5/100	95	5
	99/1/100	99	1
	96/4/100	96	4
	99/1/100	99	1

x= missing data. Group 1 = stored, but not transported control. Group 2 = Transport simulation for 6 hours in full container. Group 3 = Transport simulation for 36 hours in full container. Group 4 = Transport simulation for 36 hours in 3/4 filled container. Group 5 = Transport simulation for 36 hours in full container added Pluronic F-68.

K3

	Positive/negative/total number of cells counted	Percentage of positive cells	Percentage of negative cells
Group 1	18/84/100	18	84
	2/97/100	3	97
	5/95/100	5	95
	5/95/100	5	95
	11/55/66	17	83
	1/99/100	1	99
Group 2	0/100/100	0	100
	2/98/100	2	98
	15/85/100	15	85
	2/98/100	2	98
	x	x	x
Group 3	1/99/100	1	99
	2/98/100	2	98
	4/96/100	4	96
	6/94/100	6	94
	0/100/100	0	100
	23/77/100	23	77
Group 4	7/93/100	7	93
	2/98/100	2	98
	3/97/100	3	97
	0/100/100	0	100
	x	x	x
	2/98/100	2	98
Group 5	4/96/100	4	96
	4/96/100	4	96
	9/91/100	9	91
	6/94/100	6	94
	16/84/100	16	84
	0/100/100	0	100

x= missing data. Group 1 = stored, but not transported control. Group 2 = Transport simulation for 6 hours in full container. Group 3 = Transport simulation for 36 hours in full container. Group 4 = Transport simulation for 36 hours in 3/4 filled container. Group 5 = Transport simulation for 36 hours in full container added Pluronic F-68.

Ki67

	Positive/negative/total number of cells counted	Percentage of positive cells	Percentage of negative cells
Group 1	9/91/100	9	91
	16/84/100	16	84
	43/57/100	43	57
	22/78/100	22	78
	x	x	x
	45/55/100	45	55
Group 2	47/53/100	47	53
	34/66/100	34	66
	9/91/100	9	91
	44/56/100	44	56
	x	x	x
Group 3	28/72/100	28	72
	29/71/100	29	71
	17/83/100	17	83
	58/42/100	58	42
	46/54/100	46	54
	23/77/100	23	77
Group 4	37/63/100	37	63
	7/93/100	7	93
	24/76/100	24	76
	30/70/100	30	70
	x	x	x
	26/74/100	26	74
Group 5	20/80/100	20	80
	13/87/100	13	87
	27/73/100	27	73
	54/46/100	54	46
	19/81/100	19	81
	36/64/100	36	64

x= missing data. Group 1 = stored, but not transported control. Group 2 = Transport simulation for 6 hours in full container. Group 3 = Transport simulation for 36 hours in full container. Group 4 = Transport simulation for 36 hours in 3/4 filled container. Group 5 = Transport simulation for 36 hours in full container added Pluronic F-68.

PCNA

	Positive/negative/total number of cells counted	Percentage of positive cells	Percentage of negative cells
Group 1	45/65/100	45	65
	60/40/100	60	40
	80/20/100	80	20
	84/16/100	84	16
	54/46/100	54	46
	61/39/100	61	39
Group 2	75/35/110	75	35
	57/43/100	57	43
	42/58/100	42	58
	70/30/100	70	30
	46/54/100	46	54
Group 3	63/37/100	63	37
	63/37/100	63	37
	50/50/100	50	50
	73/27/100	73	27
	63/37/100	63	37
	53/47/100	53	47
Group 4	0/51/51	0	100
	52/48/100	52	48
	86/56/142	61	39
	63/37/100	63	37
	x	x	x
	36/64/100	36	64
Group 5	43/57/100	43	57
	68/32/100	68	32
	78/22/100	78	22
	69/31/100	69	31
	42/58/100	42	58
	60/40/100	60	40

x= missing data. Group 1 = stored, but not transported control. Group 2 = Transport simulation for 6 hours in full container. Group 3 = Transport simulation for 36 hours in full container. Group 4 = Transport simulation for 36 hours in 3/4 filled container. Group 5 = Transport simulation for 36 hours in full container added Pluronic F-68.

Caspase 3

	Positive/negative/total number of cells counted	Percentage of positive cells	Percentage of negative cells
Group 1	1/99/100	1	99
	0/100/100	0	100
	6/94/100	6	94
	3/97/100	3	97
	x	x	x
	0/100/100	0	100
Group 2	5/95/100	5	95
	0/100/100	0	100
	0/100/100	0	100
	2/65/67	3	97
	x	x	x
Group 3	2/98/100	2	98
	1/99/100	1	99
	4/96/100	4	96
	4/96/100	4	96
	1/99/100	1	99
	2/98/100	2	98
Group 4	3/97/100	3	97
	1/99/100	1	99
	1/99/100	1	99
	4/96/100	4	96
	5/95/100	5	95
	0/100/100	0	100
Group 5	0/100/100	0	100
	0/100/100	0	100
	0/100/100	0	100
	1/99/100	1	99
	5/95/100	5	95
	2/98/100	2	98

x= missing data. Group 1 = stored, but not transported control. Group 2 = Transport simulation for 6 hours in full container. Group 3 = Transport simulation for 36 hours in full container. Group 4 = Transport simulation for 36 hours in 3/4 filled container. Group 5 = Transport simulation for 36 hours in full container added Pluronic F-68.

Supplementary Data file S5:
Metabolic datasets underlying the analyses in the study.

Group	pH	pCO2	pO2	Na+	K+
1	7.331	3.89	25.03	134.5	4.87
1	7.335	3.8	25.19	134.6	4.86
1	7.363	3.73	26.35	133.3	4.84
1	7.42	3.09	23.8	133.4	4.82
1	7.374	3.45	28.49	133.5	4.84
1	7.336	3.86	29.14	134,00	4.85
2	7.32	4.02	24.02	134.5	4.85
2	7.34	3.77	23.66	134.7	4.85
2	7.357	3.78	25.82	133.4	4.84
2	7.359	3.78	24.07	133.4	4.83
2	7.381	3.37	27.93	133.6	4.84
2	7.339	3.79	27.25	134,00	4.84
3	7.326	3.85	24.78	134.7	134.7
3	7.338	3.65	23.34	134.8	134.8
3	7.359	3.81	24.32	133.4	133.4
3	7.342	3.84	23.12	133.4	133.4
3	7.379	3.3	23.47	133.9	133.9
3	7.333	3.86	26.21	134.1	134.1
4	7.362	3.57	24.64	134.5	134.5
4	7.402	3.02	24.46	134.8	134.8
4	7.401	3.26	24.16	133.4	133.4
4	7.379	3.48	24.06	133.3	133.3
4	7.406	3.1	25.51	133.7	133.7
4	7.368	3.43	26.32	134.5	134.5
5	7.321	3.79	24.22	131.1	131,00
5	7.317	3.85	24.11	131,00	129.6
5	7.366	3.63	24.27	129.6	131,00
5	7.34	3.8	23.76	129.9	129.9
5	7.343	3.64	25.24	130.2	130.2
5	7.33	3.73	25.28	130.8	130.8

X= Missing data. Group 1 = Non-transported control group.
Group 2 = transport simulation for 6 hours in full container.
Group 3 = Transport simulation for 36 hours in full container.
Group 4 = Transport simulation for 36 hours in 3/4 filled container.
Group 5 = Transport simulation for 36 hours in full container added Pluronic F-68.

Group	Ca++	Cl-	Glu
1	1.41	118	5.1
1	1.4	118	5.00
1	1.42	118	5.1
1	1.42	118	5.1
1	1.42	119	5.2
1	1.42	119	5.1
2	1.41	118	4.9
2	1.4	117	4.9
2	1.42	118	5.1
2	1.41	119	5.1
2	1.43	119	5.2
2	1.41	118	5.1
3	1.41	117	5.00
3	1.4	117	4.9
3	1.42	119	5.1
3	1.41	118	5.1
3	1.43	119	5.1
3	1.41	119	5.1
4	1.4	118	5.00
4	1.39	118	4.9
4	1.42	118	5.00
4	1.41	119	5.1
4	1.42	118	5.1
4	1.41	118	4.9
5	1.37	115	4.9
5	1.36	115	4.93
5	1.39	116	5.00
5	1.38	115	5.00
5	1.39	116	5.00
5	1.37	115	4.9

X= Missing data. Group 1 = Non-transported control group.
Group 2 = transport simulation for 6 hours in full container.
Group 3 = Transport simulation for 36 hours in full container.
Group 4 = Transport simulation for 36 hours in 3/4 filled container.
Group 5 = Transport simulation for 36 hours in full container added Pluronic F-68.
